# TMTC1 promotes invasiveness of ovarian cancer cells through integrins β1 and β4

**DOI:** 10.1038/s41417-023-00625-y

**Published:** 2023-05-23

**Authors:** Ting-Chih Yeh, Neng-Yu Lin, Chin-Yu Chiu, Tzu-Wen Hsu, Hsin-Yi Wu, Hsuan-Yu Lin, Chi-Hau Chen, Min-Chuan Huang

**Affiliations:** 1 Graduate Institute of Anatomy and Cell Biology, College of Medicine, National Taiwan University, Taipei, Taiwan; 2 Instrumentation Center, National Taiwan University, Taipei, Taiwan; 3 Department of Obstetrics and Gynecology, National Taiwan University Hospital, Taipei, Taiwan

**Keywords:** Ovarian cancer, Cell biology

## Abstract

Ovarian cancer is the most lethal gynecological malignancy and is characterized by peritoneal disseminated metastasis. Although O-mannosyltransferase TMTC1 is highly expressed by ovarian cancer, its pathophysiological role in ovarian cancer remains unclear. Here, immunohistochemistry showed that TMTC1 was overexpressed in ovarian cancer tissues compared with adjacent normal ovarian tissues, and high TMTC1 expression was associated with poor prognosis in patients with ovarian cancer. Silencing TMTC1 reduced ovarian cancer cell viability, migration, and invasion in vitro, as well as suppressed peritoneal tumor growth and metastasis in vivo. Moreover, TMTC1 knockdown reduced cell-laminin adhesion, which was associated with the decreased phosphorylation of FAK at pY397. Conversely, TMTC1 overexpression promoted these malignant properties in ovarian cancer cells. Glycoproteomic analysis and Concanavalin A (ConA) pull-down assays showed that integrins β1 and β4 were novel O-mannosylated protein substrates of TMTC1. Furthermore, TMTC1-mediated cell migration and invasion were significantly reversed by siRNA-mediated knockdown of integrin β1 or β4. Collectively, these results suggest that TMTC1-mediated invasive behaviors are primarily through integrins β1 and β4 and that TMTC1 is a potential therapeutic target for ovarian cancer.

## Introduction

Ovarian cancer has the highest mortality rate among gynecological malignancies [1]. Most patients are diagnosed at advanced stages when ovarian cancer tumors have metastasized extensively in the peritoneal cavity, resulting in a 5-year survival rate below 50% [2]. Therefore, a deeper understanding of the molecular mechanisms underlying ovarian cancer metastasis is required for the development of novel therapeutic agents to improve patient outcomes.

Integrins, a large family of cell surface proteins that play important roles in epithelial cell-matrix interactions, are heterodimeric molecules consisting of paired α and β subunits. In mammals, 18 α and 8 β subunits have been characterized; the combination of them forms 24 distinct integrins. The integrin-FAK pathway regulates various cellular functions, including adhesion, migration, invasion, survival, growth, and differentiation in normal as well as tumor tissues [3–8]. Moreover, integrins have a great impact on cancer metastasis; therefore, they are appealing targets for cancer therapy.

Glycosylation is the most complex post-translational modification of proteins, which determines the structure and function of numerous secreted and membrane-bound proteins. Protein O-mannosylation, a type of glycosylation conserved from fungi to mammals [9], is the addition of the mannose from dolichol-phosphate mannose (Dol-P-Man) to serine or threonine residues of nascent polypeptides in the endoplasmic reticulum (ER). One type of this reaction is catalyzed by POMT1 and POMT2 with α-dystroglycan (α-DG) as the main substrate in mammals [10, 11]. The O-Man glycans are crucial in muscle and brain development; its deficiency in α-DG causes a group of congenital muscular dystrophies. Recently, a broad spectrum of O-mannosylated proteins has been identified, including cadherins/protocadherins and plexins, which do not involve POMT1 and POMT2 [12, 13]. Transmembrane and tetratricopeptide repeat containing 1-4 (TMTC1-4) are newly identified enzymes responsible for the O-mannosylation of cadherins and protocadherins [12, 14]. The TMTC-mediated O-Man is not elongated into complex glycans found in POMT1/2-dependent O-Man glycosylation but is instead limited to single O-Man monosaccharide structures [13]. The O-mannosylation of E-cadherin is essential for its correct localization on the cell membrane, and altered O-mannosylation disturbs the assembly of adherens junctions in gastric cancer [15]. With respect to the O-mannosylation of plexins, it appears to be catalyzed by currently unknown enzymes in mammalian cells.

High *TMTC1* mRNA expression is associated with decreased survival of patients with gastric cancer [16]. The Cancer Genome Atlas (TCGA) database showed that *TMTC4* mRNA was overexpressed in prostate cancer tissues compared with normal prostate tissues [17]. Although TMTC-mediated O-mannosylation is conserved in mammalian cells and is expected to play crucial roles in cellular behaviors, the pathophysiologic roles of TMTC1-4 in cancers remain largely unknown. In this study, we observed that high TMTC1 expression was associated with poor prognosis in patients with ovarian cancer. Integrins β1 and β4 were identified as novel protein substrates of TMTC1. Moreover, TMTC1 promoted migration and invasion of ovarian cancer cells by modifying O-mannosylation and activity of integrins β1 and β4. Importantly, silencing of TMTC1 was sufficient to inhibit invasiveness and peritoneal metastasis of ovarian cancer cells. Our findings provide novel mechanistic insights into the role of TMTC1 in ovarian cancer pathogenesis.

## Materials and methods

### Cell lines and culture

Human ovarian cancer cell lines, including ES-2 (ATCC, Manassas, VA, USA), SKOV3 (ATCC, Manassas, VA, USA), and OVTW59 (a kind gift from Dr. P. L. Tong, Department of Obstetrics and Gynecology, National Taiwan University), were maintained in Dulbecco’s modified Eagle’s medium (DMEM) (Invitrogen, Grand Island, NY, USA) supplemented with 10% fetal bovine serum (Gibco, Gaithersburg, MD, USA). All cells were incubated in a humidified atmosphere of 5% CO2 and 95% air at 37 °C. All human cell lines have been authenticated using STR profiling within 1 year. All experiments were performed with mycoplasma-free cells.

### Antibody generation and immunohistochemistry

Ovarian cancer tissue microarrays (Biomax BC11115c and HOvaC154Su01) were purchased from US Biomax, Inc. (Rockville, MD, USA) for immunohistochemical staining. The tissue microarrays were incubated with an anti-TMTC1 polyclonal antibody (in-house) at 2 μg/mL at 4 °C overnight. The specific immunostaining was visualized with 3, 3-diaminobenzidine Quanto Chromogen in the UltraVision™ Quanto Detection System (Thermo Fisher Scientific, San Jose, CA, USA). All sections were counterstained with hematoxylin for 1 s. The intensity of TMTC1 expression was scored as 0 (negative), 1 (faint), 2 (moderate), and 3 (strong).

The in-house anti-TMTC1 polyclonal antibody was raised by immunizing rabbits with recombinant TMTC1. The pGEX-4T-1 plasmid with inserted *TMTC1* was constructed and transformed into *Escherichia coli* BL21 competent cells. The recombinant TMTC1 expression was induced for 16 h at 20 °C by adding 1 mM isopropyl-β-d-thiogalactoside when the A600nm value of the cultures was 0.4 to 0.5. The soluble fraction and the inclusion body were separated using a 9% SDS-PAGE gel and stained with Coomassie brilliant blue (Supplementary Fig. S1A). The recombinant TMTC1 (65.8 kDa) was transferred to a PVDF membrane and incubated with the anti-serum to purify the anti-TMTC1 polyclonal antibody. Then, the anti-TMTC1 polyclonal antibody on the PVDF membrane was eluted for immunohistochemistry (Supplementary Fig. S1B). Western blots confirmed that HA-tagged TMTC1 was detected by anti-TMTC1 polyclonal antibody (Supplementary Fig. S1C).

### Real-time RT-PCR analysis

Total RNA was isolated using the TRIzol reagent (Invitrogen, Grand Island, NY, USA), and cDNA was synthesized using the High Capacity cDNA Reverse Transcription Kit (Applied Biosystems, Carlsbad, CA, USA). The cDNA was subjected to real-time RT-PCR using primers for *TMTC1* (5'-TGTGTCAGAGGAGACCGGAT-3' and 5'-GGTTTCAGCTGGAGAGCCTT-3') or *GAPDH* (5'-TGAAGGTCGGAGTCAACGGATT-3' and 5'-CCTGGAAGATGGTGATGGGATT-3').

### MTT assay

Cell viability was determined using the 3-(4,5-dimethylthiazol-2-yl)-2,5-diphenyltetrazolium bromide (MTT) assay. In summary, 10 μl of 5 mg/ml MTT (Sigma, St. Louis, MO, USA) was added to each well in a 96-well plate and incubated at 37 °C for 3 h. Next, the MTT formazan crystals formed by the metabolically viable cells were dissolved in 100 μl of 0.01 N HCl with 10% SDS. Finally, the absorbance was measured at dual wavelengths of 550 and 630 nm using a microplate reader.

### Transfection and plasmid construction

The pRL-TK was an empty plasmid for control. TMTC1 plasmid was pRL-TK plasmid containing human full-length TMTC1 sequence. The TMTC1 and control empty pRL-TK constructs were acquired from the Biomedical Resource Core of the First Core Laboratory, College of Medicine, National Taiwan University. *TMTC1* overexpression was investigated by first transfecting ovarian cancer cells with the TMTC1/pRL-TK plasmids using Lipofectamine 3000 (Invitrogen, Grand Island, NY, USA). An empty pRL-TK plasmid was used as the mock transfection. In contrast, *TMTC1* was knocked down by transfecting the cells with 10 nM siRNA using Lipofectamine RNAiMAX (Invitrogen, Grand Island, NY, USA) for 48 h. The siRNA used was the control siRNA (5'-CAA CCU CAG CCA UGU CGA CUG GUU U-3') of medium GC content or two siRNAs oligonucleotides against *TMTC1*, siTMTC1-1 (5'-UGU CAC CUU UGG GAG CAC UGU AUU A-3') and si-TMTC1-3 (5'-ACG GUG UUU GGA GUG UGC UUG GUU U-3') (Invitrogen, Grand Island, NY, USA). In addition, siRNAs against *ITGB1* (si-ITGB1-1, 5'-CCU AAG UCA GCA GUA GGA ACA UUA U-3' and si-ITGB1-2, 5'-UGC GAG UGU GGU GUC UGU AAG UGU A-3'), *ITGB4* (si-ITGB4-1, 5'-GCC UAC UGC ACA GAC GAG AUG UUC A-3' and si-ITGB4-2, 5'-CCG GAU GCU GCU UAU UGA GAA CCU U-3') were synthesized. The pLKO/TMTC1-shRNA plasmid (TRCN147171) and non-targeting pLKO plasmids (TRCN208001) were purchased from National RNAi Core Facility (Academia Sinica, Taipei, Taiwan). The oligo sequence of pLKO/TMTC1-shRNA is CCGGCGATTACAAGAAGTTCGAGAACTCGAGTTCTCGAACTTCTTGTAATCGTTTTTTG. Target sequence of TMTC1 is CGATTACAAGAAGTTCGAGAA. Stable knockdown was performed with the shRNA construct in pLKO.1 plasmid using a lentivirus-based infection system. On the other hand, stable overexpression was performed with lentiviral transfer vector, pLAS2w.Ppuro-TMTC1-HA or pLAS2w.Ppuro, using a lentivirus-based infection system. Stable knockdown and overexpression cells were selected with 500 ng/ml puromycin for 14 days. The knockdown of *TMTC1* was confirmed by real-time RT-PCR. To establish stable overexpression of TMTC1 in cells, we utilized a lentivirus-based infection system and infected cells with pLAS2w.Ppuro-TMTC1-HA or pLAS2w.Ppuro lentiviral transfer vector. Subsequently, the cells were subjected to puromycin selection at a concentration of 500 ng/ml for a duration of 14 days to obtain the TMTC1 stable overexpressing cell population.

### Transwell migration assay and Matrigel invasion assay

Transwell migration assay was performed using chambers with 8-μm membranes (Corning Incorporated, Corning, NY, USA). The Transwell chambers were coated with Matrigel (Corning Incorporated, Corning, NY, USA) for the Matrigel invasion assay. Cells were detached and suspended in a serum-free medium and seeded on the upper chamber. A medium with 10% FBS in the lower chamber was used as the chemoattractant. After 24 h of incubation, the cells on the lower surface of the chamber were stained with crystal violet (Sigma, St. Louis, MO, USA) in 20% (v/v) methanol and counted under a microscope. The experiments were performed in triplicates, and four fields of each transwell were analyzed.

### Adhesion assay

Collagen I, collagen IV, fibronectin, laminin, or control bovine serum albumin (BSA) at 5 μg/ml in PBS were coated on 6-wells plates for 3 h and blocked with 1% BSA in PBS at 4 °C overnight. Cells (4 × 10^5^) in 1 ml serum-free DMEM medium per well were allowed to attach to coated plates at 37 °C for 20 min. Then, the adhered cells were counted manually under an inverted microscope. In addition, the cells were collected for western blot analysis.

### LC-MS/MS analysis and database search

The extracellular domain of integrins β1 and β4 expressed by HEK293 were purchased from Sino Biological for high-energy collision activated dissociation (HCD) and/or electron-transfer/higher-energy collision dissociation (EThcD) mass spectrometry. LC-MS/MS analysis with HCD and/or EThcD fragmentation was conducted on an Orbitrap Fusion Lumos Tribrid quadrupole-ion trap-Orbitrap mass spectrometer (Thermo Fisher Scientific, San Jose, CA, USA) equipped with a nanospray interface. Peptides were separated on an Ultimate system 3000 nanoLC system (Thermo Fisher Scientific, Bremen, Germany) linked to a mass spectrometer. In summary, peptides were loaded onto a 75-μm ID, 25-cm C18 Acclaim PepMap NanoLC column (Thermo Scientific, San Jose, CA, USA) packed with 2-μm particles with 100-A pores. Mass spectrometry analysis was performed in a data-dependent mode with Full-MS with a resolution of 120,000 at m/z = 200, AGC target 5e5, maximum injection time of 50 msec. HCD-MS/MS (resolution of 15,000) of the most intense ions in 3 s was used to fragment multiply charged ions within a 1.4 Da isolation window at a normalized collision energy of 32%. AGC target 5e4 was set for MS/MS analysis with previously selected ions dynamically excluded for 180 s. Max injection time 50 ms. The ETD reaction time was set to 250 ms for EThcD (resolution of 60,000). HCD supplemental activation was enabled with normalized collision energy set to 15%. (calculation based on precursor m/z and charge state). The AGC target was set to 4e5 for EThcD.

Protein identification was performed by searching the raw MS/MS data against the Uniprot human database using the Mascot search algorithms (version 2.3) via the Proteome Discoverer package (version 2.2, Thermo Scientific, San Jose, CA, USA). The mass spectrometry proteomics data have been deposited to the ProteomeXchange Consortium via the PRIDE [18] partner repository with the dataset identifier PXD037907.

### ConA pull-down assay

The changes in O-mannose were analyzed by first treating 250 μg or more of total cell lysates with PNGase F (New England Biolabs, Ipswich, MA, USA) to remove the N-glycans. Then, the cell lysates were incubated with ConA-agarose beads (Vector Laboratories, Burlingame, CA, USA) at 4 °C overnight. Next, the ConA-agarose beads were washed with PBS five times to remove unbound proteins and subjected to Western blotting. For cell surface biotinylation, OVTW59 cells were transfected with control siRNA (siControl) or TMTC1 siRNA (siTMTC1-1). After using EZ-Link Sulfo-NHS-Biotin (Thermo Fisher Scientific, San Jose, CA, USA) to biotinylate surface molecules, cell lysates were treated with PNGase F to remove N-glycans. Then, ConA-agarose beads were used to pull down glycoproteins with mannoses. The biotinylated glycoproteins were detected using streptavidin-HRP and an ECL kit.

### Western blot analysis

Cell lysates were extracted from cell pellets in NP40 buffer. The proteins were separated on a 9% SDS-PAGE gel and transferred onto a PVDF membrane. The membrane was blocked in 5% BSA (Bio-Rad, Hercules, CA, USA) at room temperature for 1 h and incubated with a primary antibody against integrin β4 (Cell Signaling Technology, Danvers, MA, USA), integrin β1 (BD Biosciences, San Jose, CA, USA), p-FAK (Cell Signaling Technology, Danvers, MA, USA), FAK (Santa Cruz Biotechnology, Santa Cruz, CA, USA), TMTC1 (in-house) or GAPDH (Santa Cruz Biotechnology, Santa Cruz, CA, USA) at 4 °C overnight. Then, the membranes were incubated with horseradish peroxidase-conjugated secondary antibodies, and the protein bands were detected using ECL reagents (GE Healthcare Life Sciences, Pittsburgh, PA, USA).

### Peritoneal metastasis assay

Female BALB/cAnN.Cg-Foxnlnu/CrlNarl nude mice aged between 5 and 7 weeks were procured from the National Laboratory Animal Center, Taiwan. They are in general SPF conditions. The nude mice were fed a normal diet LabDiet5010. The water for nude mice is sterilized reverse osmosis (RO) water at the Animal Center of National Taiwan University College of Medicine. For peritoneal injection, we used 1 mL syringes and 24 G needles. ES-2 and SKOV3 cells were mixed in a 1:1 ratio with Matrigel™ Basement Membrane Matrix (Corning Incorporated, Corning, NY, USA), and subsequently, either 5 × 10^6^ ES-2 cells or 1 × 10^7^ SKOV3 cells were injected intraperitoneally into the nude mouse. The formation of metastatic nodules was assessed by measuring their numbers and weights 15 or 40 days post-injection of ES-2 or SKOV3 cells, respectively, after which the mice were sacrificed. All animal procedures were conducted following the guidelines approved by the Animal Ethics Committee.

### Statistical analysis

Statistical analyses were performed using the SPSS 22.0 (SPSS, Chicago, IL, USA) statistical software package. The Student’s *t-*test, ANOVA and Kaplan–Meier plotter were used to analyze experiments. Data were shown as means ± SD, and two-sided *P* < 0.05 is considered statistically significant.

## Results

### TMTC1 expression is upregulated in ovarian cancer and higher TMTC1 expression is associated with poorer prognosis in patients with ovarian cancer

**Fig. 1:**
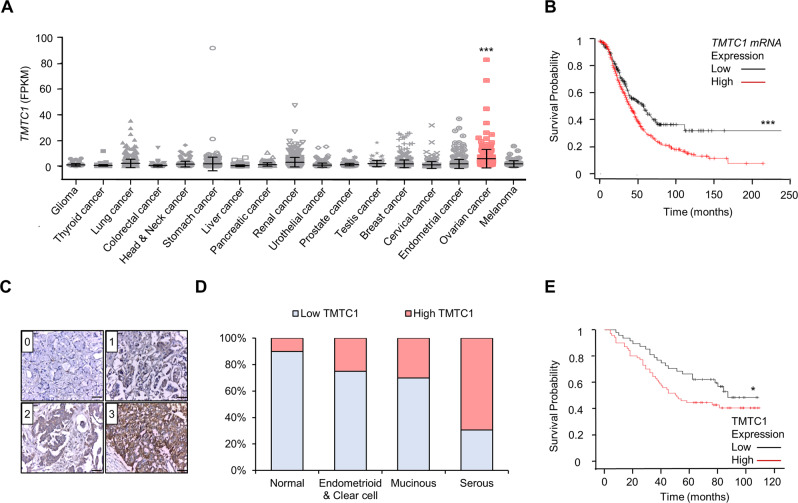
TMTC1 expression is upregulated in ovarian cancer and higher TMTC1 expression is associated with poorer prognosis in patients with ovarian cancer. **A** TMTC1 expression in various cancers from the TCGA database. The tumor tissues of 373 serous ovarian cancer patients had higher TMTC1 mRNA levels than glioma (*n* = 153), thyroid cancer (*n* = 501), lung cancer (*n* = 994), colorectal cancer (*n* = 597), head and neck cancer (*n* = 499), stomach cancer (*n* = 354), liver cancer (*n* = 365), pancreatic cancer (*n* = 176), renal cancer (*n* = 877), urothelial cancer (*n* = 406), prostate cancer (*n* = 494), testis cancer (*n* = 134), breast cancer (*n* = 1075), cervical cancer (*n* = 291), endometrial cancer (*n* = 541), and melanoma (*n* = 102). Fragments Per Kilobase of exon model per Million mapped fragments (FPKM) for each gene was used for quantification of expression with a detection threshold of 1 FPKM. Data were analyzed using one-way ANOVA. ***P < 0.001. **B** Analysis of overall survival using the Kaplan-Meier plotter. Expression ranges of the probes are from 3 to 4993. Kaplan-Meier plotter takes the probe value of 201 as the cut point between high and low expression. The median survival of low expression cohort is 57.1 months and high expression cohort is 40 months. Kaplan-Meier plotter analyzes all histological subtypes with all grades. The patient numbers for high (red) and low (black) TMTC1 expression are 444 and 211, respectively. ***P < 0.001. **C** Scoring TMTC1 levels from 0 to 3 in ovarian cancer tissues immunostained with an anti-TMTC1 antibody. Scale bar, 0.5 mm. **D** Immunohistochemistry of TMTC1 levels in ovarian tumors (*n* = 90), including 62 serous carcinomas, 10 mucinous carcinomas, 3 endometrioid carcinomas, 5 clear cell carcinomas, and adjacent normal ovary tissues (*n* = 10). **E** Kaplan–Meier survival curve for overall survival. TMTC1 expression in ovarian cancer tissue microarrays was divided into the low (0–1; *n* = 43) and high (2–3, *n* = 73) groups. The data exclude cases of specimens from metastatic sites, rare histologic types, and missing data, and include cases of 57 high-grade serous, 14 low-grade serous, 30 mucinous, 13 endometrioid, and 2 clear cell types. *P < 0.05.

Initially, to discover the expression pattern of TMTC1 in various cancer tissues, we searched TCGA database and used RNA-seq data from 17 cancer types for comparisons. We found that *TMTC1* expression levels in serous type ovarian adenocarcinomas were significantly higher than those in other tumors (Fig. 1A). We then examined the prognostic value of *TMTC1* expression in a public database Kaplan-Meier plotter (KM plotter) based on microarray data from ovarian cancer patients. The Kaplan-Meier survival analysis showed that ovarian cancer patients with high *TMTC1* expression had poorer survival (Fig. 1B). In addition, we examined TMTC1 levels in a tissue microarray (Biomax BC11115c) using immunohistochemical staining. The TMTC1 levels were scored from 0 to 3 (Fig. 1C). Samples were then classified as either low (0 and 1) or high (2 and 3) for TMTC1 protein expression. The results showed that TMTC1 was frequently overexpressed in cancerous ovarian specimens compared with normal tissues (Fig. 1D). We further used the clinical samples from another tissue microarray (Biomax HOvaC154Su01) to analyze the effect of TMTC1 on the survival of ovarian cancer patients. Consistently, the Kaplan-Meier analysis confirmed that high TMTC1 expression was significantly associated with poor overall survival (Fig. 1E). Notably, the multivariate analysis showed that in addition to lymph node and distant metastasis, TMTC1 is an independent prognostic factor for poor prognosis (*P* = 0.03) (Supplementary Table S1). These findings suggest that TMTC1 expression is elevated in cancerous ovarian tissues and that high TMTC1 expression predicts poor survival in patients with ovarian cancer.

### TMTC1 promotes malignant phenotypes in ovarian cancer cells

**Fig. 2:**
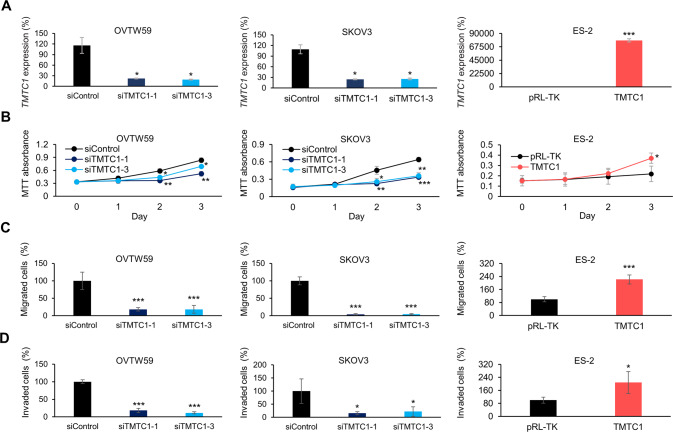
TMTC1 promotes malignant phenotypes in ovarian cancer cells. **A** TMTC1 knockdown or overexpression in ovarian cancer cells. OVTW59 and SKOV3 cells were transfected with non-targeting siRNA (siControl) or two independent siRNAs against TMTC1 (siTMTC1-1 and siTMTC1-3) for 48 h. ES-2 cells were transfected with empty pRL-TK or pRL-TK/TMTC1 plasmid for 48 h. The relative transcript levels of TMTC1 were measured by real-time RT-PCR, and the results were normalized to GAPDH mRNA levels. Representative results from three independent experiments were shown. **B** Effects of TMTC1 on cell viability. *TMTC1* was knocked down in OVTW59 and SKOV3 cells using two independent siRNAs, siTMTC1-1 and siTMTC1-3. A non-targeting siRNA (siControl) was used as the control. TMTC1 was overexpressed in ES-2 cells using pRL-TK/TMTC1 (TMTC1). An empty pRL-TK plasmid was used for control. First, 1 × 10^3^ OVTW59, SKOV3, and ES-2 cells were seeded into each well of 96-well plates. Viable cells were measured using the MTT assay at different time points, as indicated. *n* = 3. **C** Effects of TMTC1 on migration. After transfection for 48 h, 3.5 × 10^4^ OVTW59, 1 × 10^4^ SKOV3, and 1 × 10^4^ ES-2 cells were seeded for the transwell migration assay. In the lower chamber, 10% FBS was used as a chemoattractant. After incubation for 24 h, migrated cells were counted from 4 fields under an inverted microscope. Representative results from three independent experiments were shown. **D** Effects of TMTC1 on cell invasion. After transfection for 48 h, 3.5 × 10^4^ OVTW59, 1 × 10^4^ SKOV3, and 1 × 10^4^ ES-2 cells were seeded for the Matrigel invasion assay. After incubation for 24 h, invaded cells were counted. Representative results from three independent experiments were shown. Data were analyzed using the Student’s *t*-test. Data are presented as mean ± SD (*n* = 3). *P < 0.05; **P < 0.01; ***P < 0.001.

To investigate the effects of TMTC1 on malignant cell behaviors in vitro, cell viability, migration and invasion were assessed in ovarian cancer cells. First, we analyzed endogenous levels of *TMTC1* in ovarian cancer cells. SKOV3 cells had the highest *TMTC1* level followed by OVTW59 cells, whereas ES-2 cells had the lowest *TMTC1* level (Supplementary Fig. S2). Therefore, we chose SKOV3 and OVTW59 cells for *TMTC1* knockdown experiments and ES-2 cells for *TMTC1* overexpression experiments. *TMTC1* knockdown or overexpression was confirmed using real-time RT-PCR analysis (Fig. 2A). The average knockdown efficiencies of TMTC1 in OVTW59 and SKOV3 cells were 79.2% and 75.1%, respectively. The overexpression of TMTC1 in ES-2 cells was 762.5 times compared with the control. The results of MTT assays indicated that *TMTC1* knockdown decreased, whereas *TMTC1* overexpression increased, cell viability (Fig. 2B). The transwell migration assay showed that knockdown of *TMTC1* decreased migration in both OVTW59 and SKOV3 cells, whereas *TMTC1* overexpression increased migration in ES-2 cells (Fig. 2C). The Matrigel invasion assay indicated that TMTC1 knockdown suppressed invasion in OVTW59 and SKOV3 cells (Fig. 2D). By contrast, TMTC1 overexpression enhanced ES-2 cell invasion. Moreover, TMTC1 knockdown decreased migration and invasion in ES-2 cells, which is consistent with the findings in SKOV3 and OVTW59 cells (Supplementary Fig. S3). These findings suggest that TMTC1 promotes the viability, migration and invasion of ovarian cancer cells.

### TMTC1 enhances cell-laminin adhesion in ovarian cancer cells

**Fig. 3:**
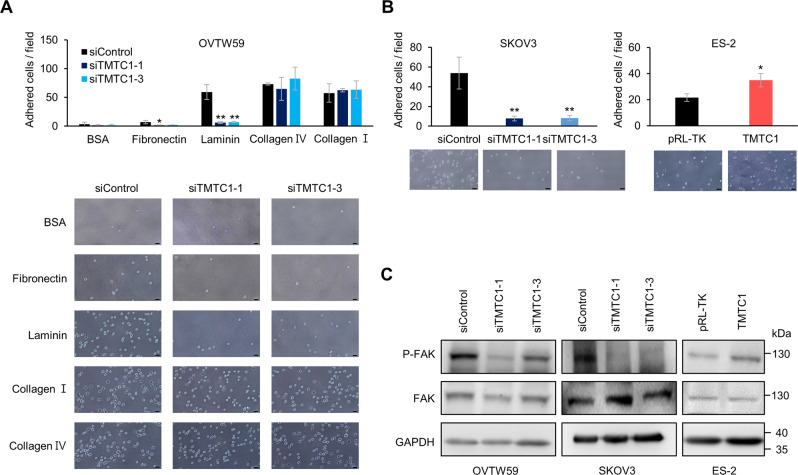
TMTC1 enhances cell-laminin adhesion in ovarian cancer cells. **A** Cell-ECM adhesion assay. TMTC1 knockdown OVTW59 cells were seeded into 6-well plates coated with 5 μg/mL laminin, collagen I, collagen IV, or fibronectin; BSA was used as a control. After 20 min of incubation at 37 °C, adhered cells were counted in 4 fields under a microscope. Representative images were shown at the lower panel. Representative results from three independent experiments were shown. Data were analyzed using the Student’s *t*-test. *P < 0.05; **P < 0.01. **B** Cell-laminin adhesion assay. SKOV3 cells with *TMTC1* knockdown and ES-2 cells with TMTC1 overexpression were seeded into 6-well plates coated with 5 μg/mL laminin. Adhered cells were counted in 4 fields under a microscope. Representative images were shown at the lower panel. Representative results from three independent experiments were shown. Results are presented as mean ± SD (*n* = 3). Data were analyzed using the Student’s *t-*test. *P < 0.05; **P < 0.01. **C** Effects of TMTC1 on laminin-mediated tyrosine phosphorylation of FAK. OVTW59 and SKOV3 cells with *TMTC1* knockdown and ES-2 cells with TMTC1 overexpression were plated onto culture plates coated with 5 μg/mL of laminin in serum-free DMEM. Changes in FAK phosphorylation (pY397) were analyzed using Western blotting. GAPDH was an internal control.

Given that adhesion ability is involved in migration and invasion [19], we next investigated whether TMTC1 modulated cell adhesion to extracellular matrix (ECM) proteins, including fibronectin, laminin, collagen I, and collagen IV. The results showed that *TMTC1* knockdown significantly decreased cell adhesion to laminin in OVTW59 cells (Fig. 3A). Moreover, *TMTC1* knockdown also decreased cell-laminin adhesion in SKOV3 cells. In contrast, *TMTC1* overexpression increased cell-laminin adhesion in ES-2 cells (Fig. 3B). Since cell-laminin adhesion enhances FAK phosphorylation, we analyzed FAK phosphorylation at pY397 in ovarian cancer cells seeded on laminin-coated plates. We quantified the pFAK and total FAK from Western blots in ovarian cancer cells. The results showed that *TMTC1* knockdown decreased, whereas TMTC1 overexpression increased, FAK phosphorylation (Supplementary Fig. S4). As expected, *TMTC1* knockdown decreased FAK phosphorylation while OVTW59 and SKOV3 cells adhered to laminin (Fig. 3C). In contrast, TMTC1 overexpression enhanced FAK phosphorylation in ES-2 cells. These results suggest that TMTC1 enhances cell-laminin adhesion and the downstream FAK signaling pathway in ovarian cancer cells.

### TMTC1 modifies O-mannosylation of integrins β1 and β4 in ovarian cancer cells

**Fig. 4:**
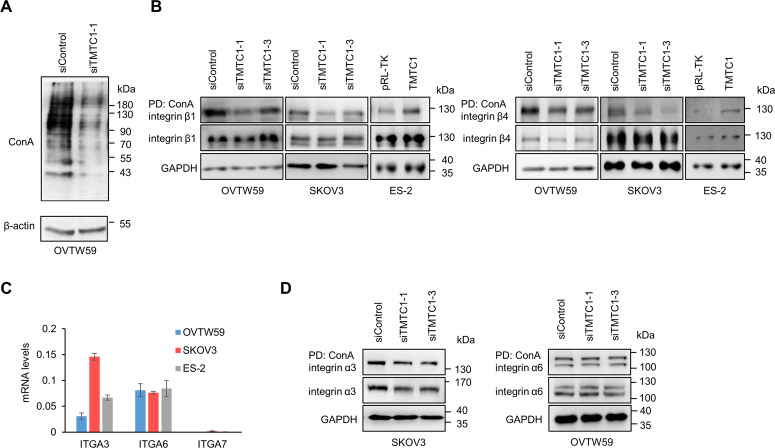
TMTC1 modifies O-mannosylation of integrins β1 and β4 in ovarian cancer cells. **A** ConA pull-down assay. OVTW59 cells were transfected with control siRNA (siControl) or *TMTC1* siRNA (siTMTC1-1). After surface biotinylation, cell lysates were treated with PNGase F to remove N-glycans. Then, ConA-agarose beads were used to pull down glycoproteins with mannoses. The biotinylated glycoproteins were detected using streptavidin-HRP and an ECL kit. **B** ConA pull-down assay of integrin β1 and β4. *TMTC1* was knocked down with siRNAs in OVTW59 and SKOV3 cells. TMTC1 was overexpressed in ES-2 cells. Cell lysates were treated with PNGase F and pulled down with ConA-agarose beads. GAPDH was used as a loading control. **C** The mRNA levels of ITGA3 (integrin α3), ITGA6 (integrin α6), and ITGA7 (integrin α7) in OVTW59, SKOV3, and ES-2 cells were determined using real-time RT-PCR analysis. The results were normalized to GAPDH mRNA levels. Data are presented as mean ± SD (*n* = 3). **D** ConA pull-down assay of integrin α3 and α6. SKOV3 cells were transfected with control siRNA (siControl) or two different TMTC1 siRNA (siTMTC1-1 or siTMTC1-3). The cell lysates were treated with PNGase F and then pulled down with ConA-agarose beads. integrin α3 was analyzed using Western blotting. GAPDH was used as loading control. OVTW59 cells were transfected with control siRNA (siControl) or TMTC1 siRNA (siTMTC1-1 or siTMTC1-3).

To unravel the underlying mechanism of TMTC1-mediated phenotypic changes in ovarian cancer cells, we identified the protein substrates of TMTC1 using a glycoproteomic approach. Because ConA can bind N-glycans with glucoses and mannoses, cell lysates were treated with PNGase F to remove the N-glycans. Thus, TMTC1-mediated changes in O-Man can be detected by performing the ConA pull-down assay with control or TMTC1 knockdown cells. We found that *TMTC1* knockdown in OVTW59 cells decreased the amount of cell surface proteins pulled down by the ConA-agarose beads (Fig. 4A), suggesting that ConA can recognize TMTC1 protein substrates after the removal of N-glycans. Next, we used ConA-agarose beads to pull down the PNGase F-treated proteins in OVTW59 cells transfected with control or *TMTC1* siRNA and then performed LC-MS/MS analysis. The results uncovered 17 proteins in the secretory pathway with decreased ConA binding (>2-fold change) in TMTC1 knockdown cells (Supplementary Table S2). Cadherins/protocadherins are known for TMTC1-4 protein substrates. Our MS proteomics data showed that ConA binding to protocadherin 7 was slightly decreased to 0.87 fold in TMTC1 knockdown cells (Supplementary Table S3). The O-mannosylation site in protocadherin 7 was confirmed to be S656 of peptide 650-ENLQPN’’S’’PVGMVTVMDADKGR-670 (Supplementary Fig. S5). Moreover, the BioPlanet database showed that the functional pathways of the 17 proteins were most closely related to integrin family cell surface interactions, followed by support of platelet aggregation by Eph kinases and ephrins (Supplementary Table S4).

Our data showed that TMTC1 enhanced cell-laminin adhesion. Among the potential protein substrates of TMTC1, integrins β1 and β4 are laminin receptors [20]. To confirm the existence of O-mannosylation on integrins β1 and β4, we analyzed their extracellular domains expressed by HEK293 cells using HCD or EThcTD mass spectrometry. The results of HCD-MS/MS showed that seven O-mannosylated sites, including S224, S263, S327, T333, S468, S474, and S587/S594, were identified in integrin β1 (Supplementary Fig. S6). Besides, S387 was identified to be O-mannosylated on integrin β4 using EThcD–MS/MS analysis (Supplementary Fig. S7). Next, we investigated the role of integrins β1 and β4 in TMTC1-mediated effects. As expected, we found that *TMTC1* knockdown decreased ConA binding to integrins β1 and β4 in OVTW59 and SKOV3 cells (Fig. 4B). For more information, we quantified the integrins β1 and β4 pulled down by ConA-agarose beads and their changes in ovarian cancer cells (Supplementary Fig. S8). The statistic results showed that TMTC1 knockdown indeed decreased ConA binding to integrins β1 and β4 but did not significantly alter their levels in OVTW59 cells. In contrast, *TMTC1* overexpression increased ConA binding to integrins β1 and β4 in ES-2 cells. These findings suggest that TMTC1 can modify the O-mannosylation of integrins β1 and β4 in ovarian cancer cells. We found that EPHA2 was one of potential TMTC1 protein substrates by our mass spectrometric analysis in OVTW59 cells. However, our results from human phospho-RTK array analysis showed that *TMTC1* knockdown had no significant effect on phospho-RTK levels including EPHA2 (Supplementary Fig. S9), suggesting that EPHA2 did not play an important role in the TMTC1-mediated malignant phenotypes.

Next, we examined whether the α subunits of laminin-binding integrins could be O-mannosylated by TMTC1. Four integrins α3β1, α6β1, α7β1 and α6β4 recognize laminins as their extracellular ligands [20]. We analyzed the levels of α subunits of integrins, α3, α6 and α7, in ovarian cancer cells and found that integrins α3 and α6 were expressed at much higher levels than integrin α7 (Fig. 4C). Next, we performed the ConA pull-down assay and found that *TMTC1* knockdown did not significantly affect the amount of integrins α3 and α6 recognized by ConA (Fig. 4D).

To investigate whether the TMTC1-modified O-mannosylation could modulate heterodimerization of integrin α and β, we performed co-immunoprecipitation assays in ES-2 and SKOV3 cells. We found that *TMTC1* overexpression slightly increased the association of integrin α6 and β1, whereas *TMTC1* knockdown decreased. (Supplementary Fig. S10).

A previous study on POMT2 target proteins in gastric cancer has shown the existence of a coordinated interplay between O-mannosylation and N-glycosylation pathway [15]. The transcript levels of MGAT5 and POMT2 showed an inverse relationship. Our real-time RT-PCR data showed that TMTC1 knockdown or overexpression did not significantly alter the mRNA expression of *MGAT5* (Supplementary Fig. S11).

### Integrins β1 and β4 play a role in the TMTC1-mediated migration and invasion of ovarian cancer cells

**Fig. 5:**
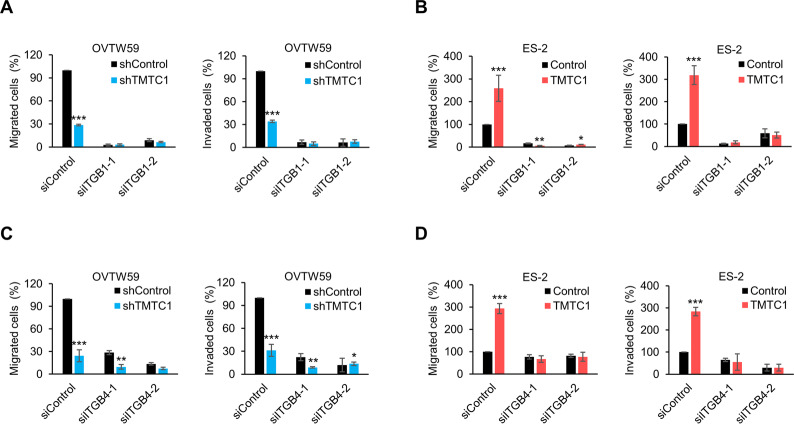
Integrins β1 and β4 play a critical role in the TMTC1-mediated migration and invasion of ovarian cancer cells. **A** Effects of *TMTC1* knockdown on cell migration and invasion were significantly blocked by *ITGB1* siRNAs in OVTW59 cells. OVTW59 cells stably transfected with the control (shControl) or *TMTC1* knockdown shRNA (shTMTC1) were transiently transfected with *ITGB1* siRNAs (siITGB1-1 and siITGB1-2). Migration and invasion of these cells were analyzed using the Transwell migration assay and Matrigel invasion assay, respectively. *n* = 3. **B** TMTC1-increased migration and invasion were significantly reversed in ES-2 cells by *ITGB1* siRNAs. The ES-2 cells stably transfected with the control (Control) or TMTC1 overexpression vector were transiently transfected with *ITGB1* siRNAs (siITGB1-1 and siITGB1-2). *n* = 3. **C** Effects of *TMTC1* knockdown on cell migration and invasion were significantly blocked in OVTW59 cells by *ITGB4* siRNAs. The OVTW59 cells stably transfected with the control (shControl) or *TMTC1* knockdown (shTMTC1) shRNA were transiently transfected with *ITGB4* siRNAs (siITGB4-1 and siITGB4-2). *n* = 3. **D** TMTC1-increased migration and invasion in ES-2 cells were significantly reversed by *ITGB4* siRNAs. ES-2 cells stably transfected with the control (Control) or TMTC1 overexpression vector (TMTC1) were transiently transfected with *ITGB4* siRNAs (siITGB4- and siITGB4-2). *P < 0.05; **P < 0.01; ***P < 0.001. Results are presented as mean ± SD (*n* = 3). Data were analyzed using the Student’s *t*-test.

Next, we investigated the role of integrin β1 or integrin β4 in the TMTC1-regulated phenotypes of ovarian cancer cells. Specifically, integrin β1 or integrin β4 was knocked down using two independent siRNAs in OVTW59 cells with TMTC1 knockdown or ES-2 cells with TMTC1 overexpression (Supplementary Fig. S12). The results showed that shRNA-mediated TMTC1 knockdown inhibited migration and invasion of OVTW59 cells (Fig. 5A). ITGB1 siRNAs dramatically suppressed migration and invasion in both control and TMTC1 knockdown cells, indicating that integrin β1 plays a critical role in OVTW59 migration and invasion. Moreover, we observed that the difference in the ability of migration and invasion between control and TMTC1 knockdown was decreased when these cells were treated with ITGB1 siRNAs. We next examined the effect of ITGB1 siRNAs on the migration and invasion of control and TMTC1 overexpressing ES-2 cells. The results showed that ITGB1 siRNAs dramatically suppressed migration and invasion in both control and TMTC1 knockdown cells (Fig. 5B). Notably, the increase of TMTC1-mediated migration and invasion was almost completely blocked. Together with the finding that TMTC1 can regulate cell-laminin adhesion and FAK activity (Fig. 3), these results suggest that integrin β1 is involved in the TMTC1-mediated migration and invasion in ovarian cancer cells. To evaluate the contribution of integrin β4 in TMTC1-mediated migration and invasion, we performed similar experiments as described above except using ITGB4 siRNAs. The results showed that ITGB4 siRNAs could also suppress migration and invasion in both control and TMTC1 knockdown cells (Fig. 5C), indicating that integrin β4 also plays an important role in OVTW59 migration and invasion. The difference in the ability of migration and invasion between control and TMTC1 knockdown was decreased by ITGB4 siRNAs (Fig. 5C). Moreover, the increase of TMTC1-mediated migration and invasion was almost completely blocked by ITGB4 siRNAs in ES-2 cells (Fig. 5D). These results suggest that integrin β4 is also involved in the TMTC1-mediated migration and invasion in ovarian cancer cells. Taken together, integrins β1 and β4 are needed for the TMTC1-mediated migration and invasion of ovarian cancer cells.

### TMTC1 promotes peritoneal tumor growth and metastasis in vivo

**Fig. 6:**
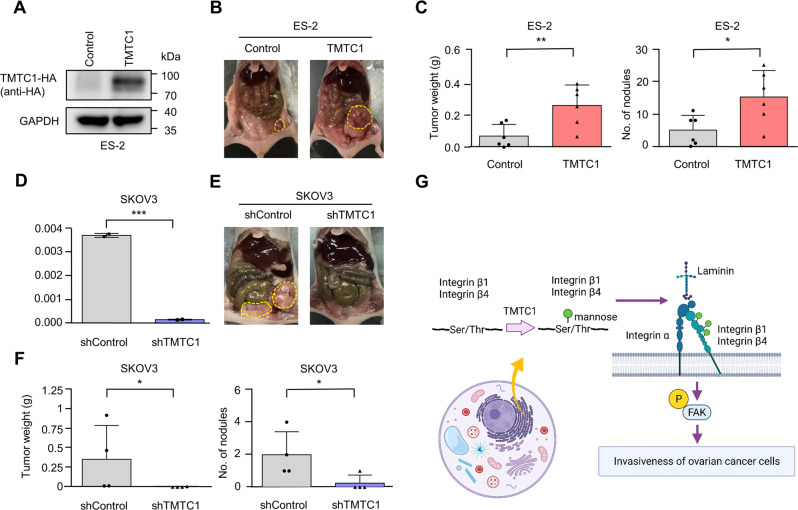
Effects of TMTC1 on tumor growth and metastasis in vivo. **A** Establishment of *TMTC1*-overexpressing ES-2 cells. HA-tagged TMTC1 was pulled down using HA tag agarose and then immunoblotted with an anti-HA antibody. **B** Representative images of tumor formation (yellow circles) in nude mice intraperitoneally injected with control or *TMTC1*-overexpressing ES-2 cells. **C** Statistical analyses of tumor weights and nodule numbers from b. Mice were sacrificed after injection of cells for 15 days. *n* = 6 for each group. *P < 0.05; **P < 0.01. Data were analyzed using the Student’s *t*-test. **D** *TMTC1* knockdown in SKOV3 cells. The relative transcript levels of *TMTC1* in stable transfectants of SKOV3 cells were measured by real-time RT-PCR and the results were normalized to *GAPDH* mRNA levels. Representative results from three independent experiments were shown. **E** Representative images of tumor formation (yellow circles) in nude mice intraperitoneally injected with the control and *TMTC1* knockdown SKOV3 cells. **F** Statistical analyses of tumor weights and nodule numbers from d. Mice were sacrificed after injection of cells for 40 days. *n* = 4 for each group. Data were analyzed using the Mann-Whitney *U* test or one-tailed Student’s *t*-test. *P < 0.05. **G** A schematic diagram showing the proposed mechanism by which TMTC1 promotes invasiveness of ovarian cancer cells. TMTC1 modifies O-mannosylation of integrins β1 and β4, increasing laminin binding and FAK phosphorylation to promote invasiveness of ovarian cancer cells. This figure was created with BioRender software.

Peritoneal dissemination is the most frequently metastatic pattern of ovarian cancer [21]. Thus, we assessed the effect of TMTC1 on peritoneal tumor growth and metastasis. First, we established stable transfectants of ES-2 cells overexpressing TMTC1 with an HA tag (Fig. 6A). Then, these ES-2 cells were injected into nude mice intraperitoneally. The results showed that TMTC1 overexpression increased tumor weights and the number of tumor nodules in the peritoneal cavity (Fig. 6B, C). Furthermore, we established stable transfectants of SKOV3 cells using *TMTC1* shRNA and injected these cells into nude mice intraperitoneally. TMTC1 knockdown was confirmed using real-time RT-PCR analysis (Fig. 6D). The silencing of *TMTC1* decreased tumor weights and the number of tumor nodules in the peritoneal cavity (Fig. 6E, F). These results suggest that TMTC1 promotes peritoneal tumor growth and metastasis of ovarian cancer cells.

## Discussion

Many patients with ovarian cancer are diagnosed at an advanced stage when the disease has spread throughout the abdominal cavity [1, 2]. To improve patient survival, there is an urgent need to find new therapeutic targets to support the development of agents to inhibit invasion and metastasis. Recently, TMTC1-4 were identified as novel O-mannosyltransferases with largely unknown protein substrates other than cadherins and protocadherins. Public databases showed that *TMTC1* mRNA is highly expressed in ovarian cancer. Here, we showed that TMTC1 protein is overexpressed in ovarian cancer and that high expression levels of TMTC1 are associated with poor prognosis. TMTC1 promotes ovarian cancer cell growth and invasiveness in vitro as well as enhances peritoneal growth and metastasis in vivo. Mechanistically, TMTC1 modulates the O-mannosylation and activity of integrins β1 and β4 to enhance cell migration and invasion (Fig. 6G). These findings suggest that TMTC1 is a potential therapeutic target and advance our understanding of TMTC1-mediated O-mannosylation in the pathogenesis of ovarian cancer.

We found that TMTC1 modifies the O-mannosylation of integrins β1 and β4 and their downstream signaling molecule p-FAK. Moreover, the TMTC1-mediated invasiveness of ovarian cancer cells was significantly reversed by siRNAs of integrin β1 or β4. Integrins β1 and β4 are frequently upregulated in ovarian cancer and promote ovarian tumorigenesis and cancer progression [7, 22, 23]. Our results showed that TMTC1 enhances cell adhesion to laminin and to a lesser extent fibronectin. Moreover, TMTC1 could slightly modulate the heterodimerization of integrin α6 and β1. Within the integrin family of cell adhesion receptors, integrins α3β1, α6β1, α6β4, and α7β1 belong to the laminin-binding subfamily [24]. Previous evidence suggested that integrin α6β1 and α6β4 play important roles in ovarian cancer invasion [25, 26]. Both integrins β1 and β4 are the β subunit of laminin receptors. Integrin β1 is also a part of the main fibronectin receptor α5β1 which also plays a role in ovarian cancer cell invasion [27]. These results strongly suggest that TMTC1 promotes the invasive behavior of ovarian cancer cells primarily through integrins β1 and β4.

A broad range of glycosylation alterations in cancer cells, including N-linked and O-linked glycosylation, have been observed. Integrin activities are strongly influenced by glycans through glycosylation events and glycan-mediated interactions [28]. It is well known that N-glycans on both α and β subunits of integrins can regulate their activities to control cell adhesion and migration [28]. For example, N-glycosylation of integrin α5β1 or α6β4 promotes the complex formation with EGFR and suppresses EGFR signaling pathway for cell growth [29]. We and others have also demonstrated that GalNAc-type O-glycosylation of integrins modulates their functions [5, 8, 30].

Here, we are the first to show the existence of TMTC1-mediated O-mannosylation in integrins β1 and β4. Moreover, this modification can enhance activities of integrins β1 and β4 to promote cell-ECM adhesion. Interestingly, our data showed that TMTC1 could not significantly affect the O-mannosylation of integrins α3 and α6, the main α subunits in ovarian cancer cells, as revealed by Con A pull-down assay. This result suggests that TMTC1 exhibits substrate preference toward integrins β1 and β4. Our data indicate that O-mannosylation occurs in integrin β1 and integrin β4. We are the first to provide the MS-based evidence demonstrating the existence of O-mannosylation sites on integrins β1 and β4. It will be of great interest to further investigate the contributions of TMTC1-4 to these sites and the role of site-specific O-mannosylation in integrins.

Besides its function as an O-mannosyltransferase, TMTC1 has been proposed to regulate calcium homeostasis via interaction with SERC2Ab [31]. Live cell calcium measurements have revealed that TMTC1 overexpression reduces calcium release from the ER following stimulation. It has been reported that decreased intracellular calcium levels suppress the migration and invasion of ovarian cancer cells [32]. However, our data showed that TMTC1 overexpression promotes migration and invasion. Therefore, the TMTC1-mediated invasiveness of ovarian cancer cells is very unlikely to occur via regulation of calcium levels.

In this study, we showed that TMTC1 is overexpressed in ovarian cancer and is an independent prognostic factor for the prediction of poor outcomes in patients with ovarian cancer. TMTC1 promotes the malignant behaviors of ovarian cancer cells in vitro and in vivo. Notably, silencing of TMTC1 is sufficient to suppress peritoneal tumor growth and dissemination. These findings not only highlight the importance of TMTC1-mediated O-mannosylation in cancer biology but also suggest that TMTC1 could be a potential target for the future development of ovarian cancer theranostics.

## Supplementary information

supplementary figure

Supplementary Tables

## Data Availability

The data that support the findings of our study are available from the corresponding author upon reasonable request.
