# Supplementary material for: TMTC1 promotes invasiveness of ovarian cancer cells through integrins β1 and β4
Source: Cancer Gene Ther. 2023 May 23;30(8):1134–43. doi: 10.1038/s41417-023-00625-y (PMC10425284; doi:10.1038/s41417-023-00625-y)
Supplement: Supplementary file 1 — supplementary figure [file 41417_2023_625_MOESM1_ESM.pdf]

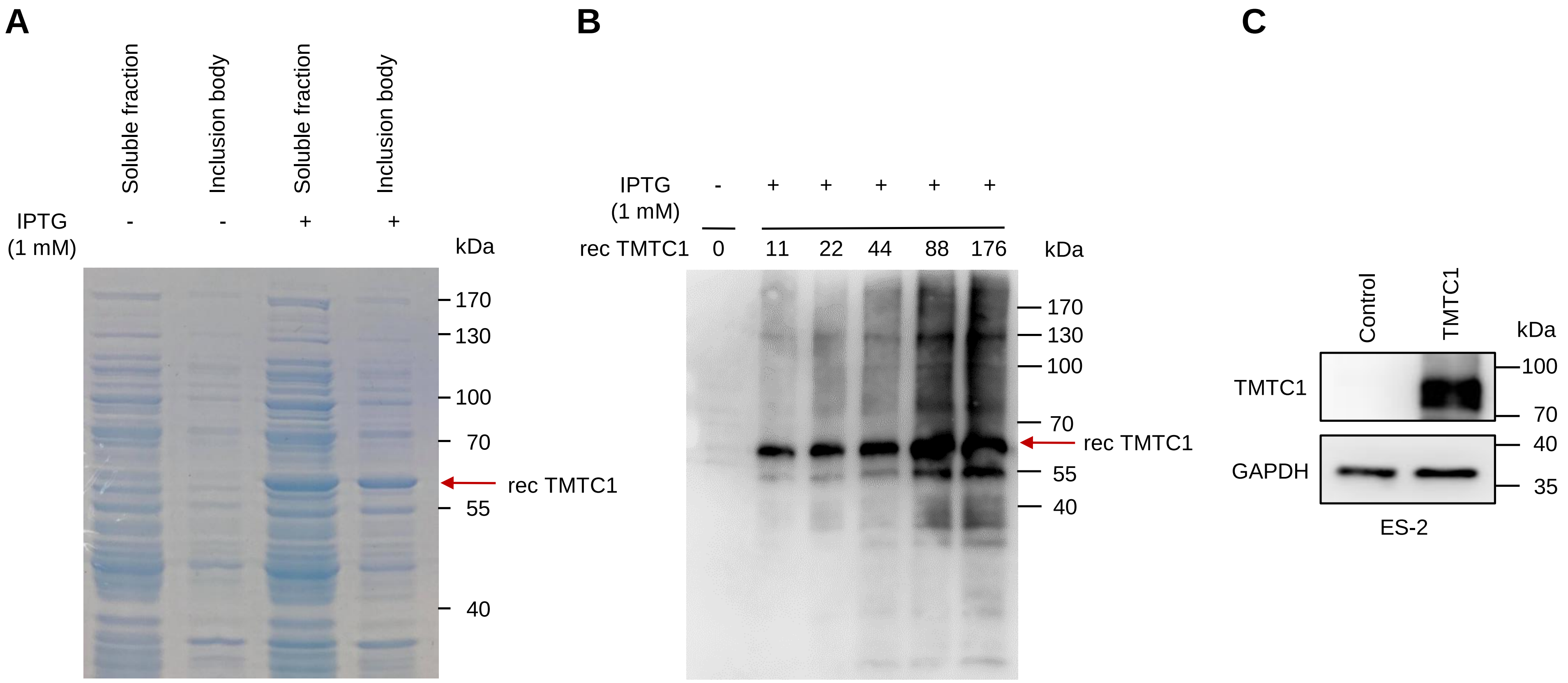

Fig. S1 Generation of an anti-TMTC1 polyclonal antibody. A SDS-PAGE gel showing the expression of recombinant TMTC1 in E. coli. Recombinant TMTC1 protein expression was induced by 1 mM IPTG at 20°C for 16 h. Proteins of E. coli lysates in the soluble fraction (1.5 µg) and the inclusion body (0.5 µg) were separated on a 9% SDS-PAGE and then stained with Coomassie brilliant blue. The red arrow indicates recombinant TMTC1 (rec TMTC1). **B** Western blot showing the specificity of anti-TMTC1 polyclonal antibody. Recombinant TMTC1 was used as an antigen to generate the polyclonal antibody against TMTC1 in rabbits. Total proteins in the inclusion bodies with estimated concentrations of recombinant TMTC1 proteins, as indicated, were separated by a 9% SDS-PAGE and then Western blotted with the affinity-purified anti-TMTC1 polyclonal antibody. The red arrow indicates recombinant TMTC1. **C** Recognition of TMTC1 overexpressed in ES-2 cells by the anti-TMTC1 polyclonal antibody. HA-tagged TMTC1 overexpressed in ES-2 cells was pulled down using HA tag-agarose and then immunoblotted with the anti-TMTC1 polyclonal antibody.

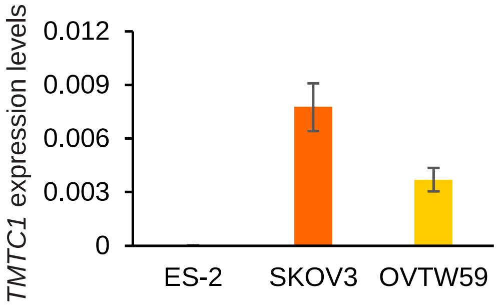

Fig. S2 Endogenous levels of *TMTC1* in ovarian cancer cells. Real-time RT-PCR analysis of *TMTC1* mRNA levels in OVTW59, SKOV3, and ES-2 cells. The results were normalized to *GAPDH* mRNA levels. Data are presented as mean ± SD.

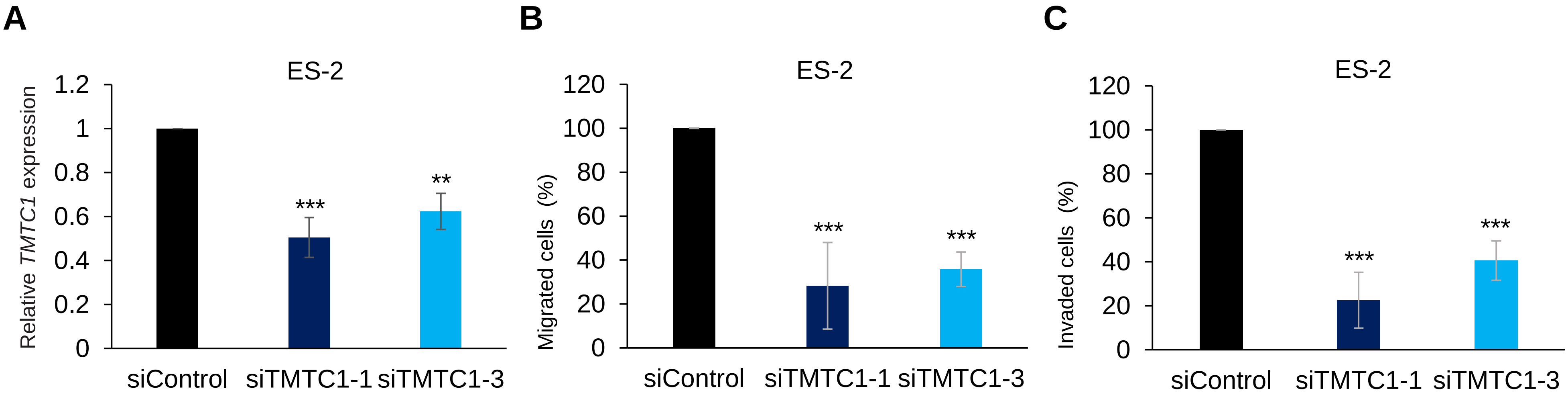

Fig. S3 TMTC1 knockdown suppresses malignant phenotypes in ES-2 cells. A TMTC1 knockdown in ES-2 cells. ES-2 cells were transfected with non-targeting siRNA (siControl) or two independent siRNAs against TMTC1 (siTMTC1-1 and siTMTC1-3) for 48 h. The relative transcript levels of *TMTC1* were measured by real-time RT-PCR, and the results were normalized to *GAPDH* mRNA levels. **B** Effects of TMTC1 on migration. After transfection for 48 h, 1×10^4 ES-2 cells were seeded for the transwell migration assay. In the lower chamber, 10% FBS was used as a chemoattractant. After incubation for 24 h, migrated cells were counted from 4 fields under an inverted microscope. **C** Effects of TMTC1 on cell invasion. After transfection for 48 h, 1×10^4 ES-2 cells were seeded for the Matrigel invasion assay. After incubation for 24 h, invaded cells were counted. Data are presented as mean ± SD. $^{**}P < 0.01$; $^{***}P < 0.001$

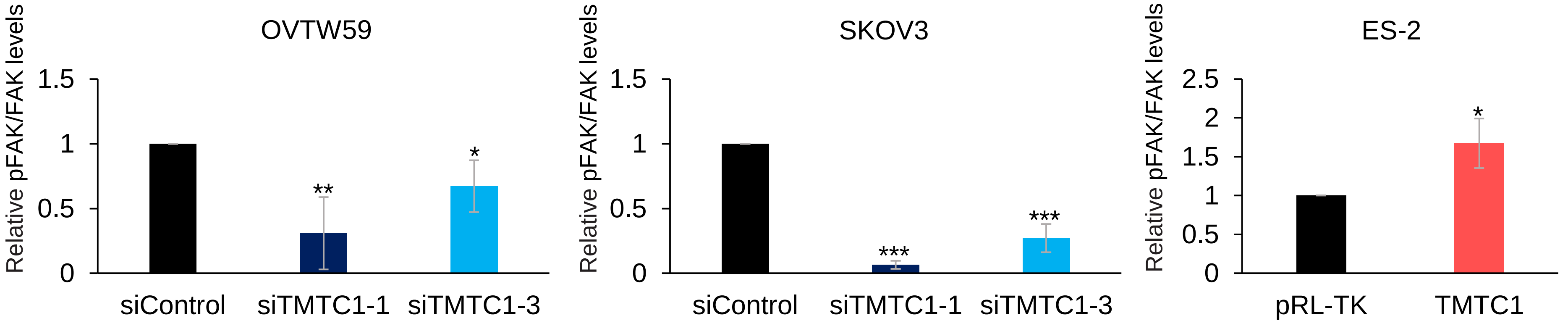

Fig. S4 Quantification of pFAK/FAK levels in ovarian cancer cells. Relative signal intensities of pFAK and FAK on Western blots in Fig. 3C were quantified by Visionworks. The ratio of pFAK/FAK was shown. **P < 0.01 ; **P < 0.01; ***P < 0.001.

Sequence: ENLQPNSPVGMVTVMDADKGR
Ions Score:41

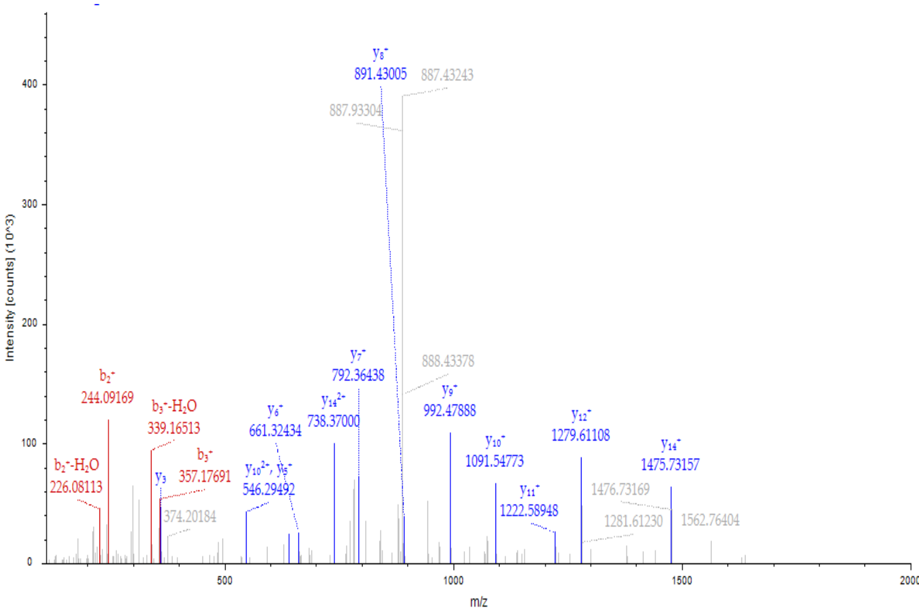

Fig. S5 The O-mannosylation site on Protocadherin 7 in OVTW59 cells. Mapping of O-mannosylation site on 650-ENLQPNSPVGMVTVMDADKGR-670 of protocadherin 7 using HCD fragmentation during MS/MS analysis. The ion y14+ suggests that S656 is O-mannosylated. The ion score of this peptide is 41.

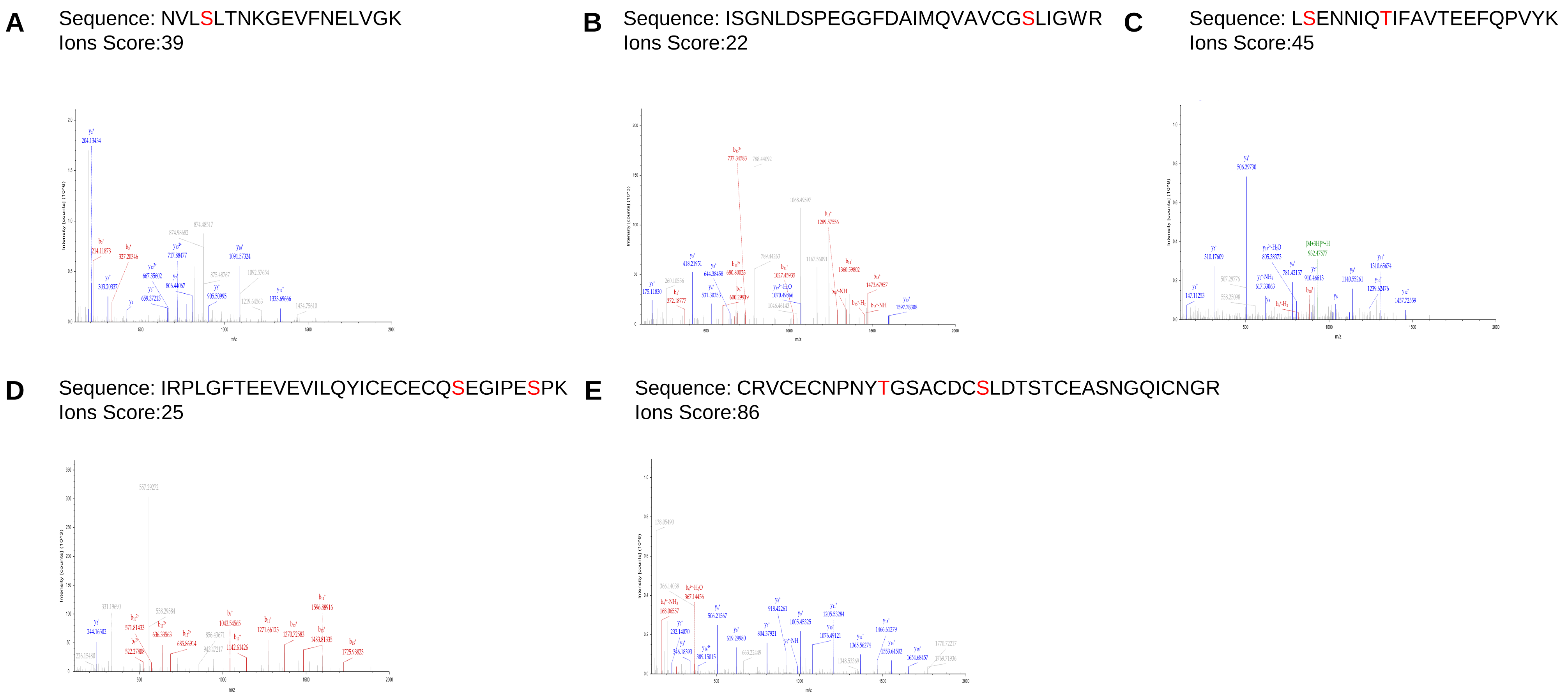

Fig. S6 The O-mannosylation sites on integrin β1. The integrin β1 from HEK293 cells was subjected to HCD-MS/MS. **A** The ions, b3+ and y142+ suggest that S224 is O-mannosylated on NVLSLTNKGEVFNELVGK. **B** The ions b15+ and y5+ suggest that S263 is O-mannosylated on ISGNLDSPEGGFDAIMQVAVCGSLIGWR. **C** The ion y12+ suggests that S327 and T333 are O-mannosylated on LSENNIQTIFAVTEEFQPVYK. **D** The ions, b15+ and y2+ suggest that S468 and S474 are O-mannosylated on IRPLGFTEEVEVILQYICECECQSEGIPESPK. **E** The ions b42+ and y15+ suggest that S587 or S594 is O-mannosylated on CRVCECNPNYTGSACDCSLDTSTCEASNGQICNGR. Amino acids in red are O-mannosylated.

Sequence: GLRTEVTSKMFQK
Ions Score:38

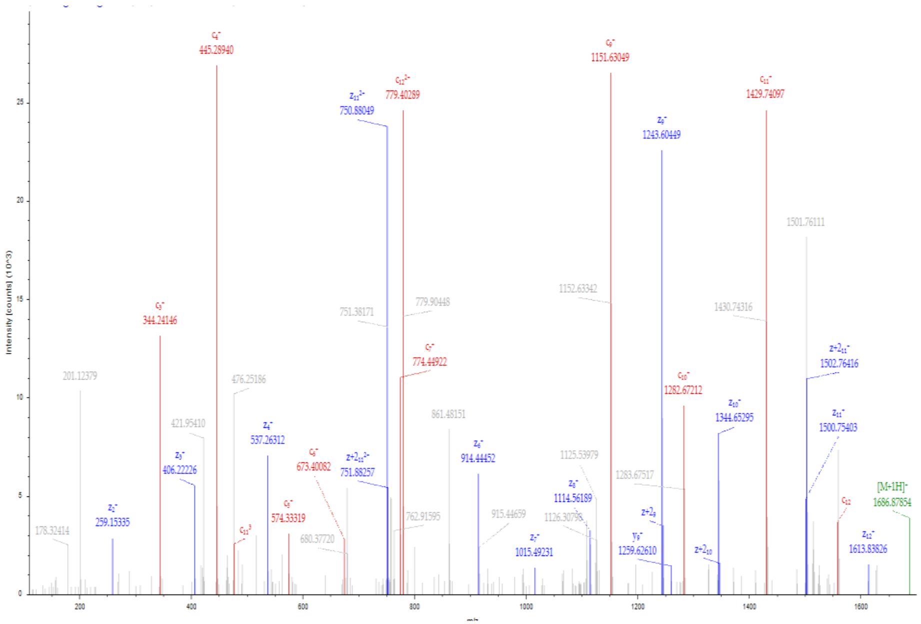

Fig. S7 The O-mannosylation site on integrin β4. Mapping of O-mannosylation site on 380-GLRTEVTSKMFQK-392 of integrin β4 from HEK293 cells using EThcD fragmentation during MS/MS analysis. The ion, c7+, c9+, z4+, and z6+ suggest that S387 is O-mannosylated. The ion score of this peptide is 38.

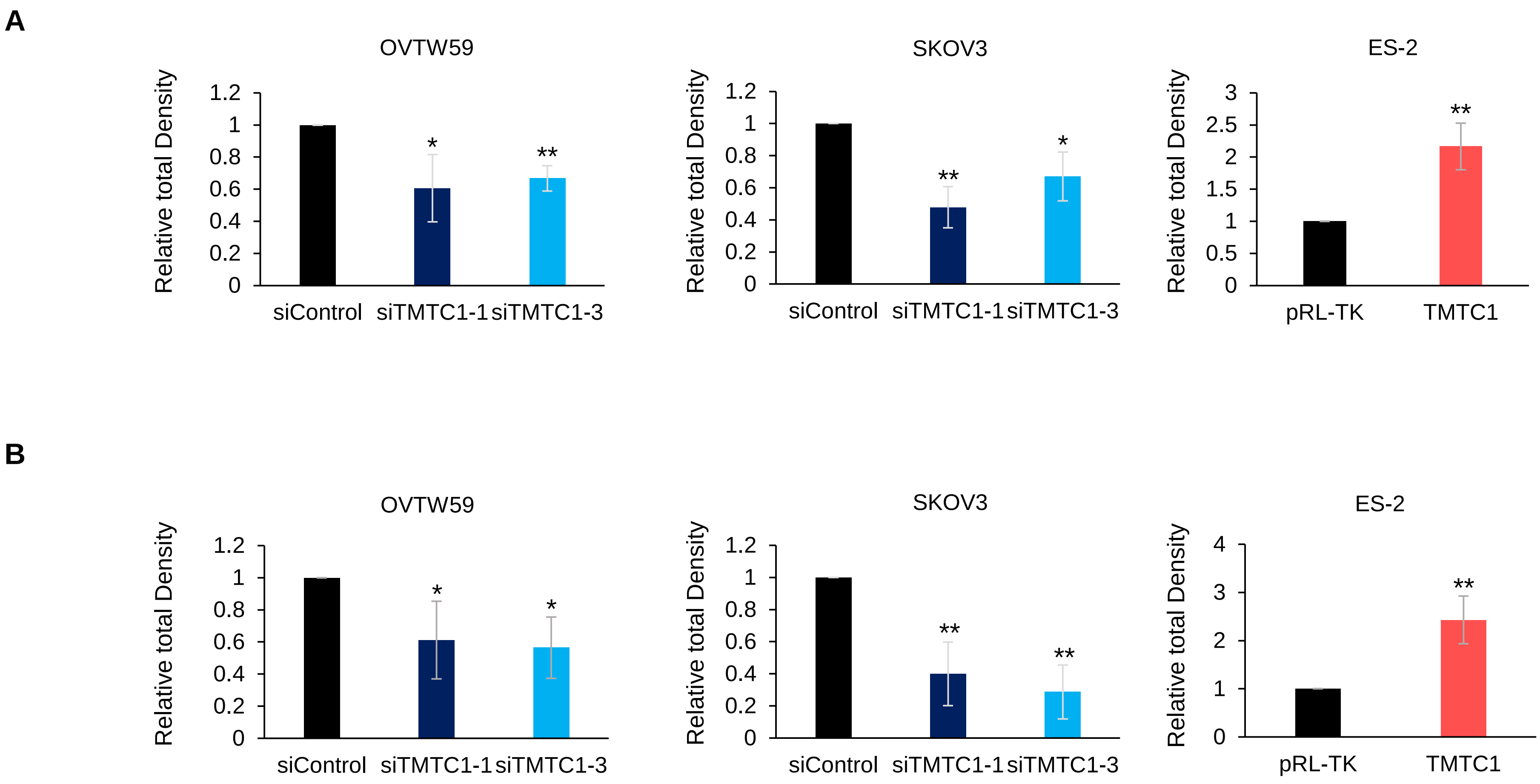

Fig. S8 Quantification of integrin β1 and β4 levels from Western blots in Fig. 4B. A Relative signal intensities of integrins β1 were quantified by Visionworks. **B** Relative signal intensities of integrins β4 were quantified by Visionworks. Data are presented as mean ± SD. *P < 0.05; **P < 0.01.

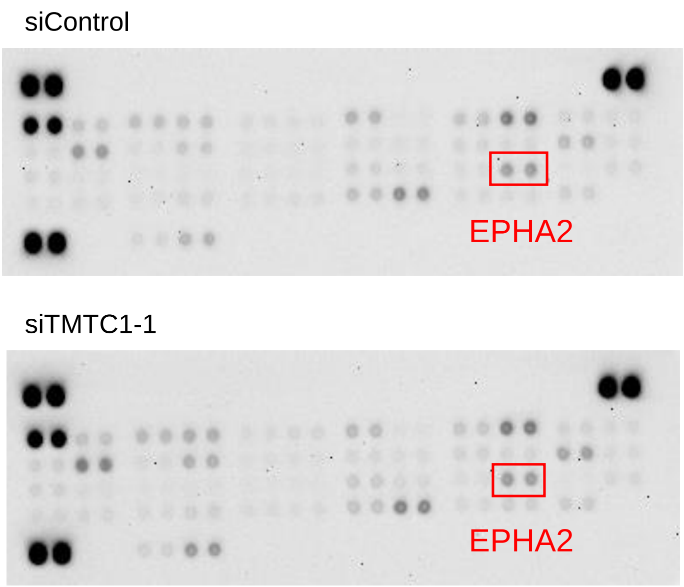

Fig. S9 Effects of TMTC1 on phospho-RTK levels. TMTC1 knockdown had no prominent effect on phospho(p)-RTK levels, including p-EPHA2 (red rectangles). OVTW59 cells was transfected with siRNA (siControl or siTMTC1-1). Cells were serum starved for 24 h and then treated with 10% FBS for 15 minutes. Phospho-RTKs were analysed by Western blotting of human phospho-RTK array (R&D Systems) according to the manufacturer’s protocol.

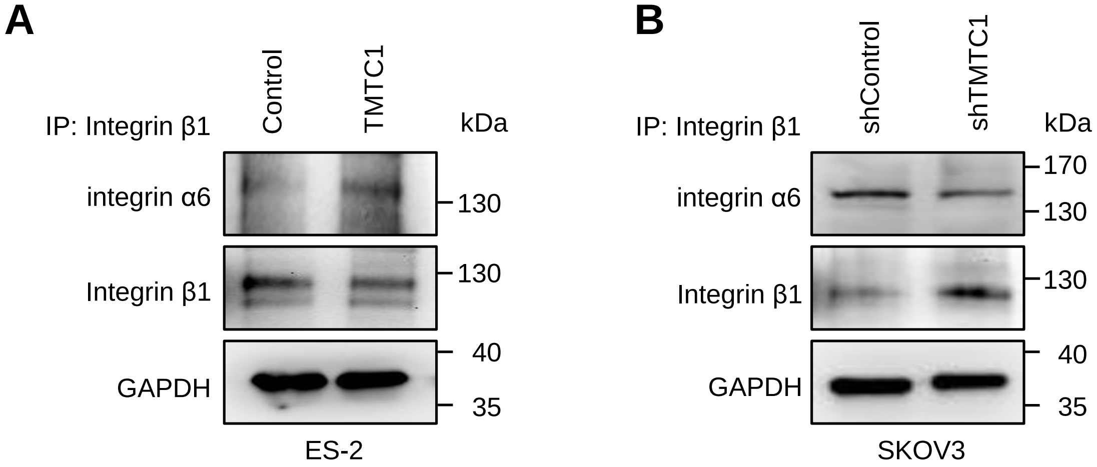

Fig. S10 TMTC1 slightly increases heterodimerization of integrin α6 and integrin β1. A Co-immunoprecipitation assay of integrin α6 and integrin β1 in ES-2 cells. Lysates from control and TMTC1-overexpressing ES-2 cells were immunoprecipitated (IP) with an anti-integrin β1 antibody and then immunoblotted with an anti-integrin α6 or anti-integrin β1 antibody. GAPDH was used as loading control. **B** Co-immunoprecipitation assay of integrin α6 and integrin β1 in SKOV3 cells.

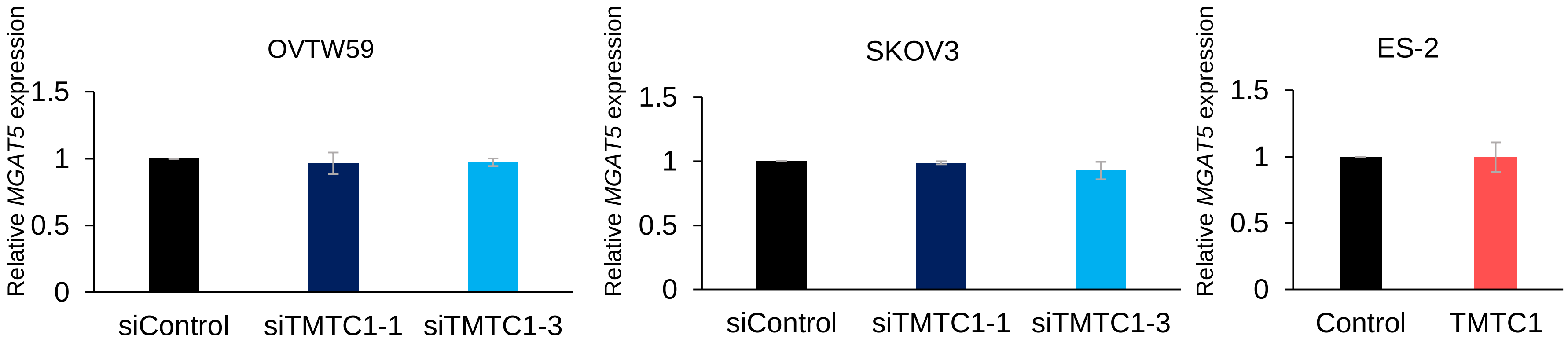

Fig. S11 TMTC1 knockdown or overexpression does not significantly alter MGAT5 transcript levels. Real-time RT-PCR analysis of *MGAT5* mRNA levels in TMTC1 knockdown OVTW59 and SKOV3 cells as well as in TMTC1 overexpressing ES-2 cells. The results were normalized to *GAPDH* mRNA levels.

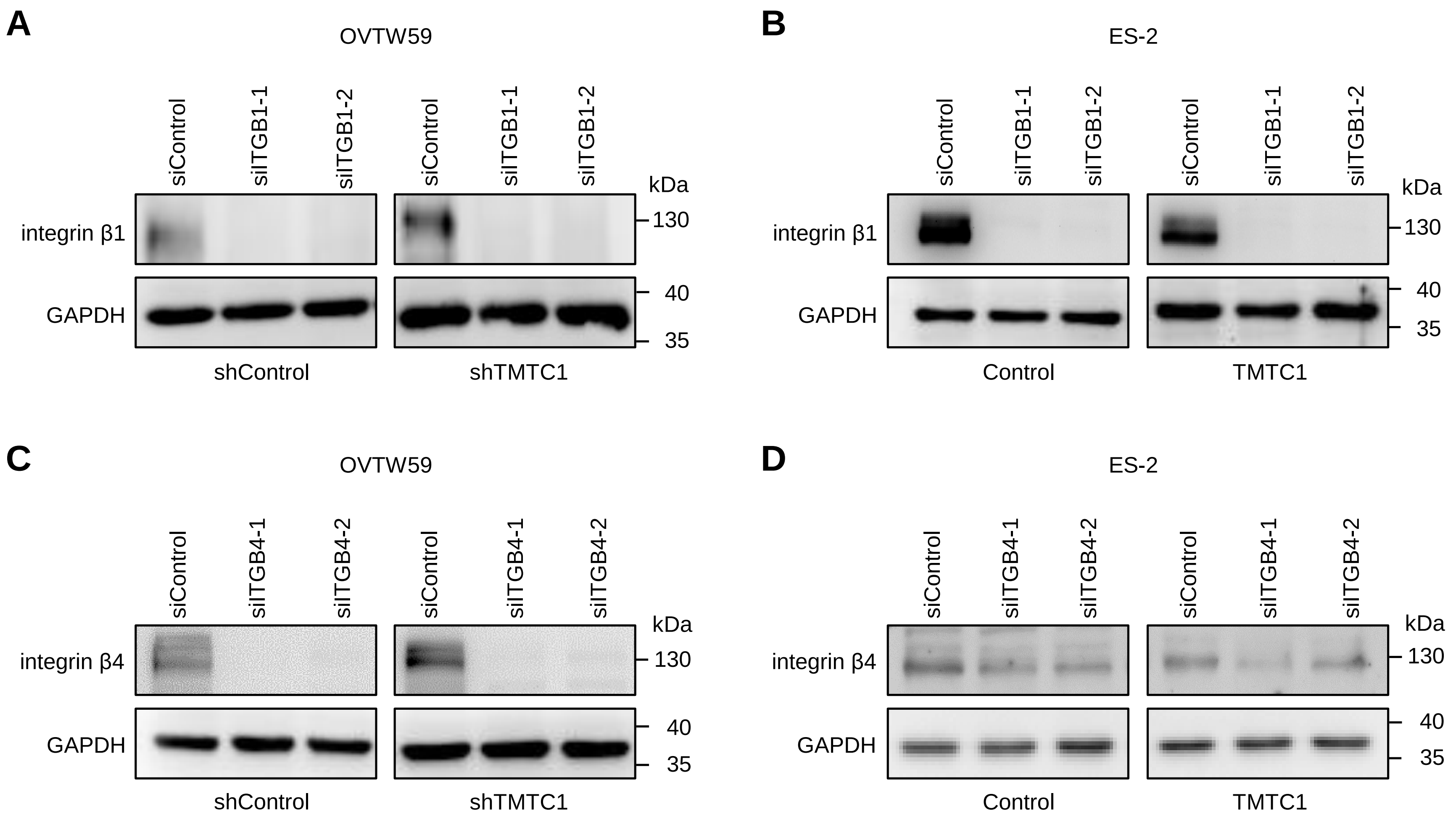

Fig. S12 ITGB1 or ITGB4 knockdown in ovarian cancer cells. A Western blots showing ITGB1 knockdown in OVTW59 cells stably transfected with the control (shControl) or TMTC1 (shTMTC1) knockdown shRNA. ITGB1 was knocked down using two independent siRNAs (siITGB1-1 and siITGB1-2). A non-targeting siRNA (siControl) was used as control. **B** Western blots showing ITGB1 knockdown in ES-2 cells stably transfected with control (Control) or TMTC1 (TMTC1) overexpression vector. **C** Western blots showing ITGB4 knockdown in OVTW59 cells stably transfected with control (shControl) or TMTC1 (shTMTC1) shRNA. ITGB4 was knocked down using two independent shRNAs (siITGB4-1 and siITGB4-2). A non-targeting siRNA (siControl) was used as the control. **D** Western blots showing ITGB4 knockdown in ES-2 cells stably transfected with control (Control) or TMTC1 (TMTC1) overexpression vector.
