# Supplementary material for: TMTC1 promotes invasiveness of ovarian cancer cells through integrins β1 and β4
Source: Cancer Gene Ther. 2023 May 23;30(8):1134–43. doi: 10.1038/s41417-023-00625-y (PMC10425284; doi:10.1038/s41417-023-00625-y)
Supplement: Supplementary file 2 — Supplementary Tables [file 41417_2023_625_MOESM2_ESM.pdf]

Table S1. Univariate and multivariate analysis of prognostic factors for overall survival in ovarian cancer patients (n=116) [a]

Variables	No.[b]	Univariate		Multivariate	
		**HR (95% CI) [c]**	***p*-value**	**HR (95% CI) [c]**	***p*-value**
**Age**					
≤50	52	1	0.615	1	0.668
>50	64	1.14 (0.69–1.90)		0.89 (0.52–1.52)	
**Lymph node**					
Negative	87	1	**< 0.001**	1	**0.003**
Positive	29	5.42 (3.23–9.10)		2.98 (1.45–6.13)	
**Distant metastasis**					
No	96	1	**< 0.001**	1	**0.002**
Yes	20	6.62 (3.72–11.79)		3.46 (1.57–7.60)	
**Histologic type**					
Serous	71	1		1	
Non-serous	45	0.58 (0.33–0.99)	**0.046**	1.06 (0.58–1.91)	0.861
**TMTC1**					
Low	43	1	**0.050**	1	**0.030**
High	73	1.72 (1.00–2.95)		1.89 (1.06–3.35)	

[a] Fourteen, 8, and 8 cases were excluded from the data of tissue microarray (Biomax HOvaC154Su01) due to specimen from metastatic sites, rare histologic types, and missing data, respectively. Data included 57 high-grade serous, 14 low-grade serous, 30 mucinous, 13 endometrioid, and 2 clear cell types.

[b] Number of patients

[c] HR, hazard ratio (CI, confidence interval) of univariate or multivariate Cox regression analysis

Table S2. Potential TMTC1 protein substrates identified by LC-MS/MS analysis in OVTW59 ovarian cancer cells.

Gene names	Control (Normalized abundances)	TMTC1 KD [a] (Normalized abundances)	Subcellular location	Ratio (TMTC1/ Control)
PRXL2A	761946.9	0	Secreted	0
EPHA2	308396.5	0	plasma membrane	0
ITGB1	305463.1	0	plasma membrane	0
ATP2B1	3158574.0	0	plasma membrane	0
ITGB4	11560705.0	621522.5	plasma membrane	0.053762
DSG1	1761521.0	198764.4	plasma membrane	0.112837
S100A9	1551257.0	198481.3	Secreted	0.127949
FABP5	3143134.0	437938.6	Secreted	0.139332
PDCD6IP	1328523.0	204573.8	Secreted	0.153986
IDE	914204.0	201437.4	Secreted	0.220342
DCD	3794587.0	933768.9	Secreted	0.246079
ANXA2	8351029.0	2352683.0	Secreted	0.281724
MYOF	32236635.0	10363300.0	plasma membrane	0.321476
LGALS7	1263902.0	416478.5	Secreted	0.329518
CLIC1	2559495.0	982493.0	plasma membrane	0.383862
ARL6IP5	692811.9	268395.8	plasma membrane	0.387401
NCEH1	1095194.0	477185.5	plasma membrane	0.435709

[a]KD, knockdown

Table S3. Protocadherin-7 identified by LC-MS/MS analysis in OVTW59 ovarian cancer cells.

**Gene names**	**Control (Normalized abundances)**	**TMTC1 KD [a] (Normalized abundances)**	**Ratio (TMTC1 KD/ Control)**
PCDH7	19395773.21	16928202.03	0.872778

[a]KD, knockdown

Table S4. Top 10 pathways for potential TMTC1 protein substrates analyzed using BioPlanet database.

Index	Name	*P*-value	Adjusted *p*-value	Odds Ratio	Combined score
1	Integrin family cell surface interactions	0.0002184	0.02337	110.88	934.67
2	Support of platelet aggregation by Eph kinases and ephrins	0.006781	0.05244	178.36	890.65
3	CHL1 interactions	0.006781	0.05244	178.36	890.65
4	Alpha-4 beta-7 integrin signaling	0.007625	0.05244	156.05	760.96
5	Type I hemidesmosome assembly	0.007625	0.05244	156.05	760.96
6	Reduction of cytosolic calcium levels	0.008469	0.05244	138.71	661.82
7	Monocyte and its surface molecules	0.009313	0.05244	124.83	583.76
8	Apoptotic cleavage of cell adhesion proteins	0.009313	0.05244	124.83	583.76
9	Integrin beta-4 pathway	0.009313	0.05244	124.83	583.76
10	Platelet adhesion to exposed collagen	0.01100	0.05563	104.02	469.12
